# Death comes

**DOI:** 10.1017/S1478951525101296

**Published:** 2025-12-29

**Authors:** Miguel Julião

**Affiliations:** Cuidados Paliativos, Equipa Comunitária de Suporte em Cuidados Paliativos PalCo, Amadora, Sintra, Portugal

death comes softly, an exhale,
a whispered breeze that bids goodbye.
not cruel, nor cold, but calm and still,
a gentle pause, a final smile.
in fading light, the soul takes flight,
beyond the dark, into some light.
no fear to scare, no pain to hold,
just peace and love left far behind.
for dying is a quiet door,
to something greater, forevermore.
a journey where the heart can rest,
in endless calm, forever blessed.

